# A hierarchical Bayesian entry time realignment method to study the long-term natural history of diseases

**DOI:** 10.1038/s41598-022-08919-1

**Published:** 2022-03-22

**Authors:** Liangbo L. Shen, Lucian V. Del Priore, Joshua L. Warren

**Affiliations:** 1 Department of Ophthalmology, University of California San Francisco, San Francisco, CA USA; 2 Department of Ophthalmology and Visual Science, Yale University School of Medicine, 40 Temple Street, Suite 1B, New Haven, CT 06510 USA; 3 Department of Biostatistics, Yale School of Public Health, 350 George Street, New Haven, CT 06511 USA

**Keywords:** Huntington's disease, Retinal diseases, Experimental models of disease, Statistics, Biomarkers, Diseases, Medical research

## Abstract

A major question in clinical science is how to study the natural course of a chronic disease from inception to end, which is challenging because it is impractical to follow patients over decades. Here, we developed BETR (Bayesian entry time realignment), a hierarchical Bayesian method for investigating the long-term natural history of diseases using data from patients followed over short durations. A simulation study shows that BETR outperforms an existing method that ignores patient-level variation in progression rates. BETR, when combined with a common Bayesian model comparison tool, can identify the correct disease progression function nearly 100% of the time, with high accuracy in estimating the individual disease durations and progression rates. Application of BETR in patients with geographic atrophy, a disease with a known natural history model, shows that it can identify the correct disease progression model. Applying BETR in patients with Huntington’s disease demonstrates that the progression of motor symptoms follows a second order function over approximately 20 years.

## Introduction

The natural history of a disease is defined as the natural course of a disease from its inception, through various clinical stages, to a point where the patient is cured, chronically disabled, or dead in the absence of treatment^1^. Understanding the natural history of a disease is crucial to patient counseling and is a prerequisite for designing clinical trials to evaluate treatment efficacy^2,3^. To date, the natural history of most acute diseases have been well defined by directly following patients in observational clinical studies^4^. However, accurate descriptions of the long-term natural history of many chronic diseases are still lacking because it is implausible to follow hundreds or thousands of patients over several decades. Existing natural history cohort studies of chronic diseases are often limited by loss to follow-up, relatively short follow-up durations, irregular time intervals between visits, inadequate number of visits, and varying durations of disease at the time of enrollment across different patients^2^.

Instead of directly following patients over decades, an alternative approach is to develop a statistical method that can infer the long-term natural history of a disease using data from patients followed over short periods. At the time of enrollment into a typical observational clinical study, patients are usually at different time points of a disease course (i.e., different entry times). By following patients with different entry times over short durations, we can theoretically collect disease progression information at various disease stages. If we can estimate the duration of disease for individual patients reliably via a statistical method, we can realign individual patients’ datasets to reconstruct the entire course of a disease. We termed this approach as entry time realignment^5^. We previously developed an entry time realignment algorithm and applied it to investigate the long-term natural history of several ophthalmological diseases, including neovascular age-related macular degeneration (AMD)^6–9^, geographic atrophy (GA) secondary to non-exudative AMD^5,10–14^, Stargardt disease^15^, and choroideremia^16,17^. Other groups have also developed and applied similar data realignment methods in various fields, including Alzheimer’s disease^18^, fibrosing interstitial lung disease^19^, drusen secondary to AMD^20^, quantitative fluorescence microscopy^21^, and video sequence reconstruction^22^. Although the algorithms developed in these studies vary and have different names, they share a common concept in realigning the entry times of different datasets to reconstruct a longer disease process^5–20^ or improve the imaging temporal resolution^21,22^. Popular long-term disease progression models in the literature include polynomial (e.g., first and second order)^10,15,20,23^ and exponential models^16,18,23^.

Although the previous entry time realignment methods have contributed significantly to the understanding of long-term natural history of many diseases and natural processes, these methods share important limitations. First, most methods utilize an approach to shift individual patient entry times iteratively until an objective score or function (e.g., R^2^, root-mean-square deviation, or a self-developed score) is optimized, which assumes no variation in the disease progression rates of individual patients^5–19,21,22^. Although these methods can infer the long-term natural history model of a disease in an “average” patient, they cannot estimate patient-level disease progression parameters reliably because the disease progression rate tends to vary widely among patients with different clinical characteristics (e.g., demographics, genetics, environmental risk factors). Second, previous entry time realignment methods either cannot compare different long-term progression models^18–22^ or rely on complicated subsequent statistical analyses to compare different models^10,15,16^. Finally, making valid inference on the model parameters is complicated using these methods because it is difficult to correctly characterize uncertainty using deterministic algorithms.

**Figure 1:**
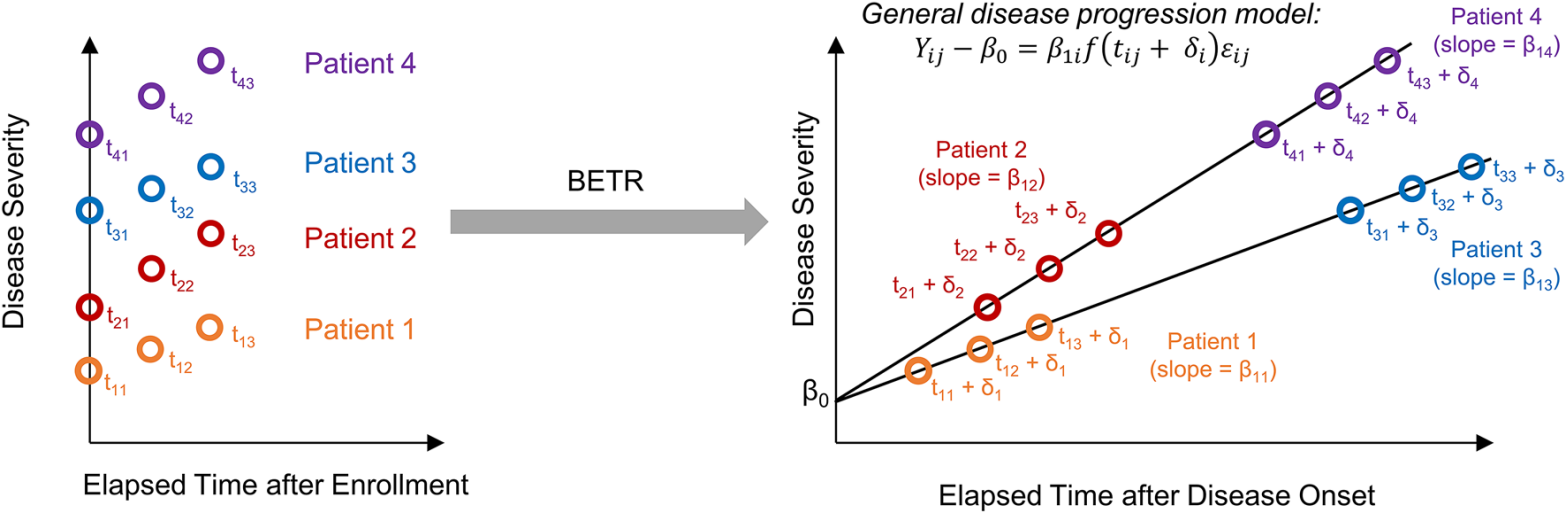
Overview of Bayesian entry time realignment (BETR) approach. Variables ${Y}_{ij}$ and ${t}_{ij}$ represent the disease severity and continuous time after enrollment into a study for patient *i* at visit *j*, respectively. ${\beta }_{0}$ represents the disease severity at the time of disease onset, which is assumed to be the same for all patients with the same disease; ${\beta }_{1i}$ is the disease progression rate for patient *i*; ${\delta }_{i}$ is the duration of disease at study enrollment for patient *i*. ${\varepsilon }_{ij}$ is the measurement error of disease severity for patient *i* at visit *j*. $\mathrm{f}(t)$ represents the disease progression model, which can be in different functional forms (e.g., linear, quadratic, exponential etc.). At the time of study enrollment (left figure), the 4 patients have different disease severities presumably because they entered the study at different time points of their individual disease courses (i.e., different ${\delta }_{i}$). By applying BETR with a hypothesized disease progression model, we can estimate ${\beta }_{1i}$, ${\delta }_{i}$, and ${\varepsilon }_{ij}$ for each patient and then horizontally realign the entry times of the patients by a factor of ${\delta }_{i}$ onto the hypothesized model (right figure). If the hypothesized disease progression model is correct, the model should fit the datasets well with a low deviance information criterion and high intraclass correlation coefficient. In this way, we can infer the long-term natural history of a disease based on datasets with short follow-up durations. In this demonstration, ${\beta }_{11}$ = ${\beta }_{13}$ and ${\beta }_{12}$ = ${\beta }_{14}$, and the correct disease progression model is the first order model.

To overcome the limitations of previous entry time realignment methods, we developed BETR (Bayesian entry time realignment), a novel hierarchical Bayesian regression modeling framework to investigate the long-term natural history of diseases based on patient data collected over short durations (see Fig. 1 for an overview). Through formal model comparisons, BETR can identify the most probable underlying long-term disease progression model among several hypothesized options with high accuracy. Additionally, BETR allows us to estimate the patient-specific disease progression parameters (i.e., disease progression rates and duration of disease at enrollment) needed to reconstruct the long-term natural course of a disease. Because BETR represents a fully specified statistical model, we can correctly quantify uncertainty in each step of the analysis, resulting in valid statistical inference for these parameters. We tested BETR using simulated data, a dataset of patients with GA (a chronic ophthalmic disease with a known long-term natural history model), and a dataset of patients with Huntington’s disease (a chronic neurological disorder with an unclear long-term natural history model).

## Results

### Simulations

**Figure 2:**
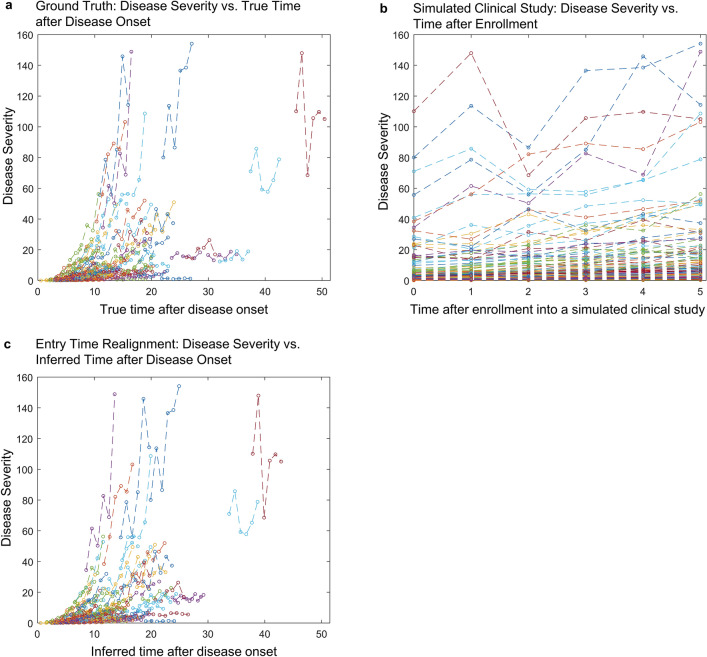
Bayesian entry time realignment (BETR) algorithm reconstructs the natural history of a disease in a simulated dataset. (**a**) Simulated natural disease progression over 5 years in 100 patients based on the second order model, serving as the ground truth. Each line represents the disease severity of an individual patient as a function of time after disease onset. The disease severity and time can have arbitrary units. In the simulation, different patients have different disease progression rates. (**b**) A simulated clinical study following the 100 patients over 5 years. In a typical clinical study, the durations of disease of individual patients are unknown so the horizontal axis is the time after enrollment into the study. (**c**) Reconstructed disease courses of the 100 patients based on applying BETR to the simulated clinical study datasets shown in (**b**).

Simulation studies show that BETR can identify the correct disease progression model (i.e., $\mathrm{f}\left(t\right)$) among competing models, and estimate patient-level disease progression parameters with high accuracy. Figure 2 shows results from one simulated dataset as an example. In this simulation setting, the disease severity at disease onset (i.e., ${\beta }_{0}$) was set to 0 and progressed following a second order model over time. Figure 2a shows simulated disease progression in 100 patients with each patient being followed annually over 5 years. Different patients had different disease progression rates (${\beta }_{1i}$) and durations of disease at the first visit (${\delta }_{i}$). Since patients’ duration of disease are typically unknown in a clinical study, the horizontal axis of the data collected from the simulated clinical study was time after enrollment into the study (Fig. 2b). Note that at enrollment, disease severity varied widely across different patients, largely because different patients entered the study at different time points of the disease natural history. After applying BETR to the simulated clinical study data shown in Fig. 2b using different hypothesized disease progression functions, we calculated the deviance information criterion (DIC) for each function. DIC is Bayesian model comparison tool that balances model fit and complexity, where a lower value indicates that the selected disease progression model is preferred^24^. We found that the second order model achieved the lowest DIC among all competing models, including first order (DIC = − 110), second order (DIC = − 186), and exponential (DIC = 39). This suggested that the second order model was the best long-term disease progression model, consistent with the predefined ground truth $\mathrm{f}\left(t\right)$. Next, we estimated ${\beta }_{1i}$ and ${\delta }_{i}$ for each patient using BETR with second order disease progression function. After horizontally translating individual datasets in Fig. 2b by a factor of ${\delta }_{i}$, the horizontal axis was changed from time after enrollment into the study to inferred time after disease onset (Fig. 2c). The reconstructed disease natural history in individual patients was comparable to the ground truth (compare Fig. 2c with Fig. 2a), suggesting that BETR was able to reconstruct approximately 30-years natural history of a disease by realigning individual datasets followed over only 5 years. The estimated ${\delta }_{i}$ were highly correlated with the true ${\delta }_{i}$, with an intraclass correlation coefficient (ICC) of 0.92 (Supplementary Fig. 1a,b). Similarly, the ICC between the estimated and true ${\beta }_{1i}$ was 0.92 (Supplementary Fig. 1c,d).

We repeated the above simulation process 100 times for each ground truth model ($\mathrm{f}(t)$). The ground truth models include: (1) first order model (different progression rates), (2) second order model (different progression rates), (3) exponential model (different progression rates), (4) first order model (same progression rates), (5) second order model (same progression rates), and (6) exponential order model (same progression rates). The functional forms of the first order, second order, and exponential models were $\mathrm{f}\left({t}_{ij}\right)={t}_{ij}$, $\mathrm{f}\left({t}_{ij}\right)={t}_{ij}^{2}$, and $\mathrm{f}\left({t}_{ij}\right)=\mathrm{exp}\left\{{t}_{ij}\right\}$, respectively. Disease progression rate (${\beta }_{1i}$) was different among different patients in models (1)–(3) (termed as “different progression rates” models) and was the same among all patients in models (4)–(6) (termed as “same progression rates” models). Note that “same progression rates” models are comparable to many previously proposed entry time realignment methods, which assumed all patients had the same progression rates^5–19,21,22^.

When the ground truth model was one of the three “different progression rates” models, BETR identified the correct disease progression model (based on DIC) in 100 out 100 simulations (Supplementary Fig. 2). Also, the “different progression rates” model had lower DIC (i.e., better performance) than the “same progression rates” model, with a mean (standard error (SE)) difference of 803 (8.16), 597 (8.32), and 173 (4.65) over 100 simulations when the ground truth was first order, second order, or exponential model, respectively. Across different ground truth models (different progression rates), the ICC between the estimated and true ${\delta }_{i}$ ranged from 0.73 to 0.90 (Table 1), with an average bias ranging from 0.19 to 1.65% (Table 1). The ICC between the estimated and true ${\beta }_{1i}$ ranged from 0.86 to 0.93, with an average bias ranging from 0.06 to 1.02% (Table 1).

**Table 1:** Performance of Bayesian entry time realignment over 100 simulations.

Ground truth model	Success rate of identifying the correct model	ICC between estimated and true ${\delta }_{i}$, mean (SE)	% Difference between estimated and true mean ${\delta }_{i}$, mean (SE)	ICC between estimated and true ${\beta }_{1i}$, mean (SE)	% Difference between estimated and true mean ${\beta }_{1i}$, mean (SE)
First order model (different progression rates)	100%	0.73 (0.01)	1.65 (1.16)	0.93 (0.01)	0.39 (0.67)
Second order model (different progression rates)	100%	0.84 (0.01)	0.33 (0.51)	0.87 (0.01)	1.02 (1.07)
Exponential model (different progression rates)	100%	0.90 (0.00)	0.19 (0.36)	0.86 (0.00)	0.04 (0.25)
First order model (same progression rates)	100%^a^	0.99 (0.00)	0.37 (0.31)	NA	− 0.16 (0.20)
Second order model (same progression rates)	100%^b^	1.00 (0.00)	− 0.02 (0.21)	NA	0.20 (0.34)
Exponential order model (same progression rates)	92%^c^	1.00 (0.00)	− 0.04 (0.31)	NA	0.19 (0.28)

*ICC* intraclass correlation coefficient, *NA* not applicable, *SE* standard error, *δ*_*i*_ baseline duration of disease, *β*_*1i*_ disease progression rate.

^a^Over 100 simulations, the entry time realignment algorithm chose the first order model (different progression rates) in 33 simulations and the first order model (same progression rates) in 67 simulations. The mean (SE) of the difference in DIC between the 2 models is 1.07 (0.48).

^b^Over 100 simulations, the entry time realignment algorithm chose the second order model (different progression rates) in 36 simulations and the second order model (same progression rates) in 64 simulations. The mean (SE) of the difference in DIC between the 2 models is 0.48 (0.36).

^C^Over 100 simulations, the entry time realignment algorithm chose the exponential model (different progression rates) in 37 simulations and the exponential model (same progression rates) in 55 simulations. In 8 simulations, the second order model (different progression rates) resulted in the lowest DIC. The mean (SE) of the difference in DIC between the exponential model (different progression rates) and the exponential model (same progression rates) is − 0.070 (0.40).

When the ground truth model was one of the three “same progression rates” models, we found that the “different progression rates” model had similar DIC as the “same progression rates” model in all three simulation settings (Supplementary Fig. 3). The mean (SE) of the difference in DIC between the two models was 1.07 (0.48), 0.48 (0.36), and − 0.070 (0.40) over 100 simulations when the ground truth model was first order, second order, or an exponential model, respectively. These results confirmed the “same progression rates” model as a subset of the “different progression rates” model, suggesting that BETR with “different progression rates” model should be used regardless of whether there is suspected variability in the disease progression rates or not. The success rate of BETR in identifying the correct disease progression model (either “same progression rates” or “different progression rates”) based on the lowest DIC was 100% when the ground truth model was first or second order model, and 92% when the ground truth model was an exponential model (Table 1). Across different ground truth models (same progression rates), the ICC between the estimated and true ${\delta }_{i}$ ranged from 0.99 to 1.00 (Table 1), with an average bias ranging from − 0.04 to 0.37% (Table 1). The bias for the estimator of ${\beta }_{1i}$ ranged from − 0.16 to 0.20 (Table 1).

### Applying BETR in studying the long-term natural history of geographic atrophy

We applied BETR to investigate the long-term progression of GA area in 318 eyes from the age-related eye disease study (AREDS). Eyes with GA have well-demarcated atrophic lesions that enlarge progressively over time^25^. The age at onset of GA in individual eyes was unknown. At the first visit, the mean ± standard deviation (SD) age of the patients was 70.2 ± 5.5 years. The mean ± SD duration of follow-up was 5.1 ± 3.0 years, ranging from 0.5 to 12 years. The mean ± SD number of visits was 5.1 ± 2.6, ranging from 2 to 12. The large variation across individual eyes made it challenging to observe the decades-long natural history of GA directly. BETR showed that the second order model (different progression rates; DIC = − 358) had lower DIC (i.e., better model performance) than competing disease progression models, including the first order model (different progression rates; DIC = − 6), the exponential model (different progression rates; DIC = 365), and the second order model (same progression rates; DIC = 1852). This was consistent with the previous finding that GA area enlarged as a second order function^10,26–30^, demonstrating the ability of BETR to identify the correct disease progression model in a real-world dataset. Additionally, the DIC results show the importance of accounting for patient-level variability in the disease progression rates. There were no obvious signs of non-convergence in the second order model (different progression rates) based on Geweke’s diagnostic^31^ and visual inspection of trace plots.

**Figure 3:**
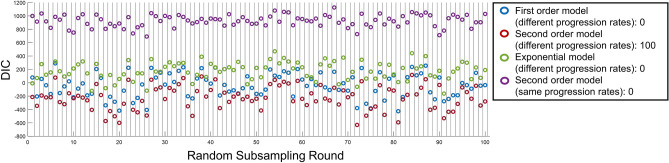
Comparing disease progression models based on the Bayesian entry time realignment (BETR) method in 100 random subsampling of 159 eyes with geographic atrophy (GA). The model with “different progression rates” means that the model assumes different progression rates among different patients and the model with “same progression rates” means that the model assumes the same progression rates among all patients. The number after the name of a model indicates the number of rounds when this model had the lowest DIC among all competing models. This figure suggests that BETR was able to identify the correct disease progression model in a real-world dataset.

Among 100 rounds of random subsampling (159 eyes per round) and refitting of BETR each time, the second order model with different progression rates had the lowest DIC among the 4 competing models in all 100 instances (Fig. 3). Note that the “same progression rates” model performed consistently worse than “different progression rates” model (Fig. 3).

**Figure 4:**
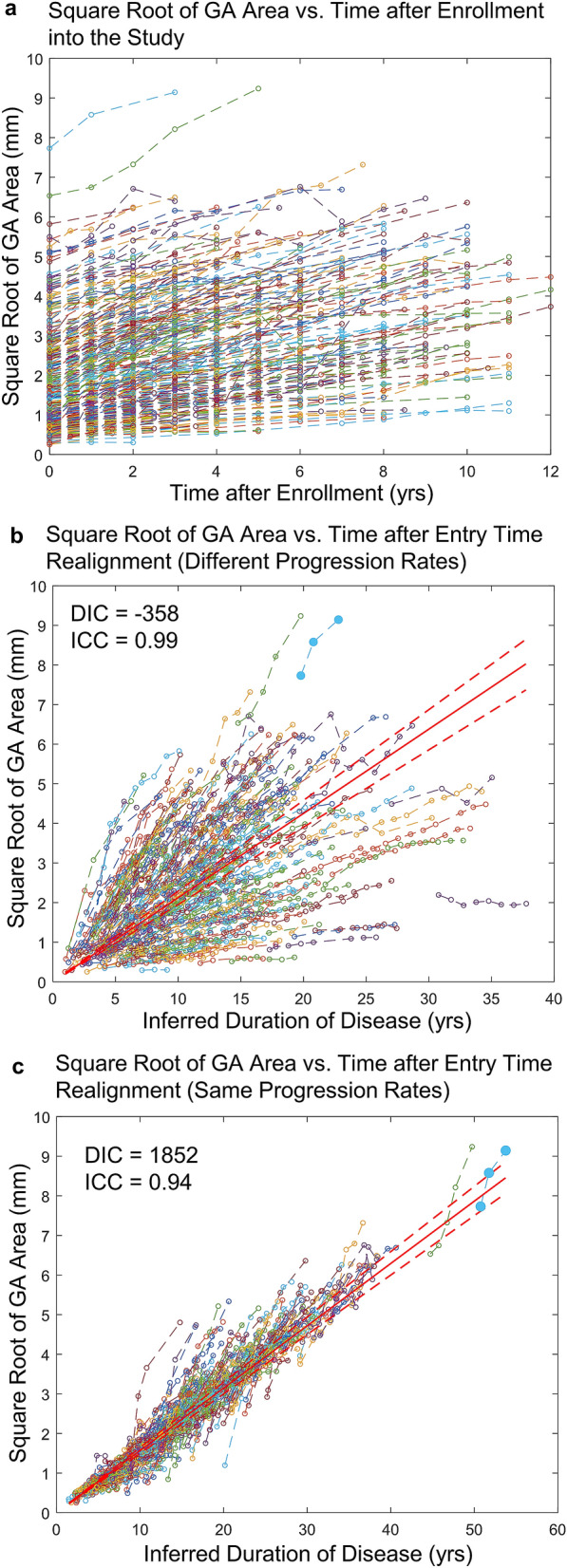
The square root of geographic atrophy (GA) area as a function of time in 318 eyes with untreated GA. (**a**) Raw GA size datasets of eyes in the Age-related Eye Disease Study. The plot shows a series of lines for GA progression in untreated eyes after enrollment into the study. Note that the initial sizes of the GA lesion varied widely among individual eyes, suggesting that different eyes had different durations of diseases at baseline and/or different progression rates. (**b**) Square root of GA area as a function of time after inferred duration of disease, which was estimated from Bayesian entry time realignment method (BETR; assuming different progression rates among different eyes). In this model, the square root of GA area in individual eyes increases linearly over time but at different rates. Note that different datasets were fitted with different trend lines in this model. The red solid line and the dashed red lines represent the mean and 95% confidence interval of the square root of GA area at each time point. (**c**) Square root of GA area as a function of time after inferred duration of disease, which was estimated from BETR (assuming same progression rates among different eyes). In this model, all datasets were fitted with the same trend line, which may be helpful to visualize the overall trend. ICC = intraclass correlation coefficient.

A linear relationship between dependent variable and independent variable is a fundamental assumption for many standard statistical methods (e.g., linear mixed model) used in clinical trials^32^. Since the enlargement of GA area followed a second order model (different progression rates), we calculated the square root of GA area and plotted it as a function of time after enrollment into the study (Fig. 4a). Note that the initial GA sizes varied widely across different patients. We estimated the duration of disease (${\delta }_{i}$) for each eye using BETR with the second order model (different progression rates). After horizontally translating datasets in Fig. 4a by a factor of estimated ${\delta }_{i}$, the horizontal axis was changed from time after enrollment to inferred duration of disease (Fig. 4b). On average, the square root of GA area enlarged linearly at a mean (SE) rate of 0.213 (0.008) mm/year over 38 years (red line in Fig. 4b). The square root of GA area enlarged linearly in individual eyes but at various rates, so datasets from individual eyes did not follow the average trend line closely (Fig. 4b). In this way, we reconstructed not only average natural history of GA (red line in Fig. 4b) but also patient-level disease course (realigned individual datasets in Fig. 4b) over decades. Based on this model, the estimated mean ± standard deviation age at onset of GA was 61.6 ± 7.3 years in this cohort. The estimated age at onset of GA in an example eye (marked in solid blue circles in Fig. 4b) was 56 years, consistent with the literature and clinical observation^33,34^. The ICC between the estimated and observed square root of GA area was 0.99 in this model, with a mean difference of − 0.002 mm (Supplementary Fig. 4a,b). The Bland–Altman plot shows that the difference between the estimated and observed square root of GA area was consistently small for values of square root of GA area, ranging from 0 to 9 mm.

To compare the “different progression rates” model and the “same progression rates” model in this dataset, we applied BETR with the second order model (same progression rates) on the same dataset and calculated ${\delta }_{i}$ for each eye based on this model. Here, we assumed that all patients had the same GA progression rate. After horizontally aligning datasets in Fig. 4a by a factor of ${\delta }_{i}$, all datasets were fit onto a single trend line (Fig. 4c). Although this plot showed the overall linear trend of the data well, the DIC of this model was very large in comparison (1852 vs. − 358), suggesting that “same progression rates” model performed much worse than the “different progression rates” model. Also, the example eye (marked in solid blue circles in Fig. 4c) had an estimated age at onset of GA of 25 years in this model, which was far earlier than the observed range of age at onset in the literature^33,34^. Additionally, the ICC between the estimated and observed square root of GA area in the “same progression rates” model was 0.92 (Supplementary Fig. 4c), lower than the “different progression rates” model (0.99). The Bland–Altman plot showed that the “same progression rates” model overestimated the square root of GA area when GA size was small and underestimated the square root of GA area when GA size was large (Supplementary Fig. 4d).

### BETR determines the long-term natural history of Huntington’s disease

We performed a similar BETR analysis to investigate the long-term progression of motor symptoms, assessed by generalized index (GI)^23^, in 71 patients with Huntington’s disease. GI score ranges from 0 (no motor features) to 100 (all motor features)^23^. The ages at onset of motor symptoms in these patients were unknown. At the first visit, the mean ± SD age of the patients was 48.6 ± 12.6 years. The mean ± SD duration of follow-up was 6.2 ± 3.0 years, ranging from 1.0 to 15.4 years. The mean ± SD number of visits was 9.1 ± 3.9, ranging from 2 to 18. Among 71 patients, 4 patients did not have data on CAG trinucleotide repeats, and the CAG repeats was less than 35 in 1 patient. BETR showed that among four competing disease progression models, the second order model (different progression rates) had the lowest DIC (DIC = 257), suggesting the second order model was the best to describe the average long-term progression of motor symptoms in Huntington’s disease. We didn’t observe any obvious signs of non-convergence in the second order model (different progression rates) based on Geweke’s diagnostic^31^ and visual inspection of trace plots.

**Figure 5:**
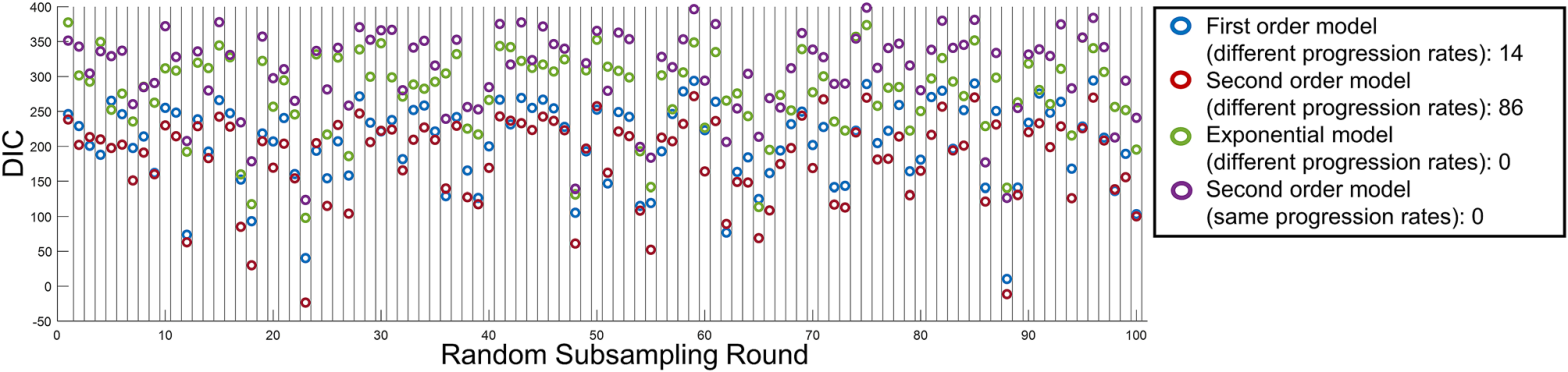
Comparing disease progression models based on the Bayesian entry time realignment method in 100 random subsampling of 50 patients with Huntington’s disease. The model with “different progression rates” means that the model assumes different progression rates among different patients and the model with “same progression rates” means that the model assumes the same progression rates among all patients. The number after the name of a model indicates the number of rounds when this model had the lowest DIC.

Among 100 rounds of random subsampling (50 patients per round), the second order model (different progression rates) had the lowest DIC in 86 rounds and the first order model (different progression rates) had the lowest DIC in 14 rounds (Fig. 5), suggesting that the progression of motor symptoms followed the second order model in most patients with Huntington’s disease. The “same progression rates” model had higher DIC (i.e., worse performance) than “different progression rates” model in all 100 subsampling rounds (Fig. 5).

**Figure 6:**
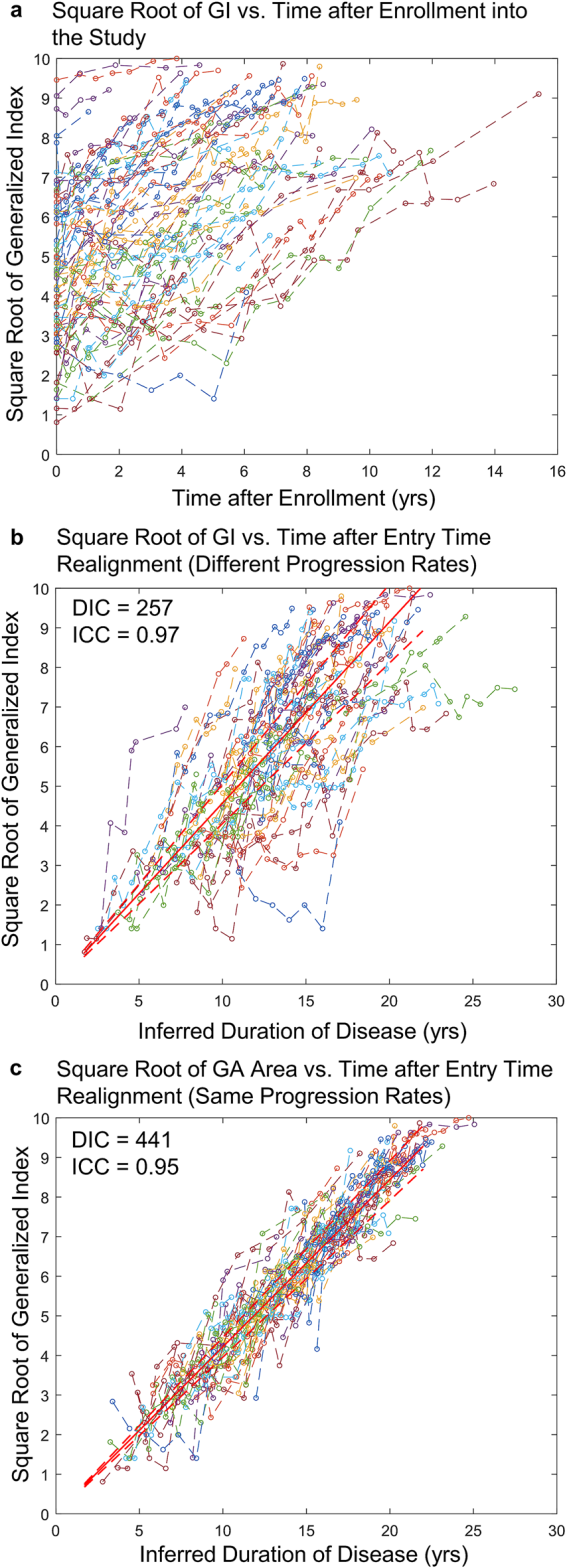
The square root of the generalized index (GI) as a function of time in 71 patients with untreated Huntington’s disease. (**a**) Raw datasets collected in the study. The plot shows a series of lines for GI progression in untreated patients with Huntington’s disease after enrollment into the study. Note that the initial GI varied widely among individual patients, suggesting that different patients had different durations of diseases at baseline; the variation in the slopes seen visually suggest there were different progression rates as well. (**b**) Square root of GI as a function of time after inferred duration of disease, which was estimated from the Bayesian entry time realignment method (BETR). In this model, the square root of GI increases linearly over time and different datasets were fit with different trend lines. The red solid line and the dashed red lines represent the mean and 95% confidence interval of the square root of GA area at each time point. (**c**) Square root of GI as a function of time after inferred duration of disease, which was estimated from BETR. In this model, all datasets were fit with the same trend line, which helpful to visualize the overall trend. ICC = intraclass correlation coefficient.

**Figure 7:**
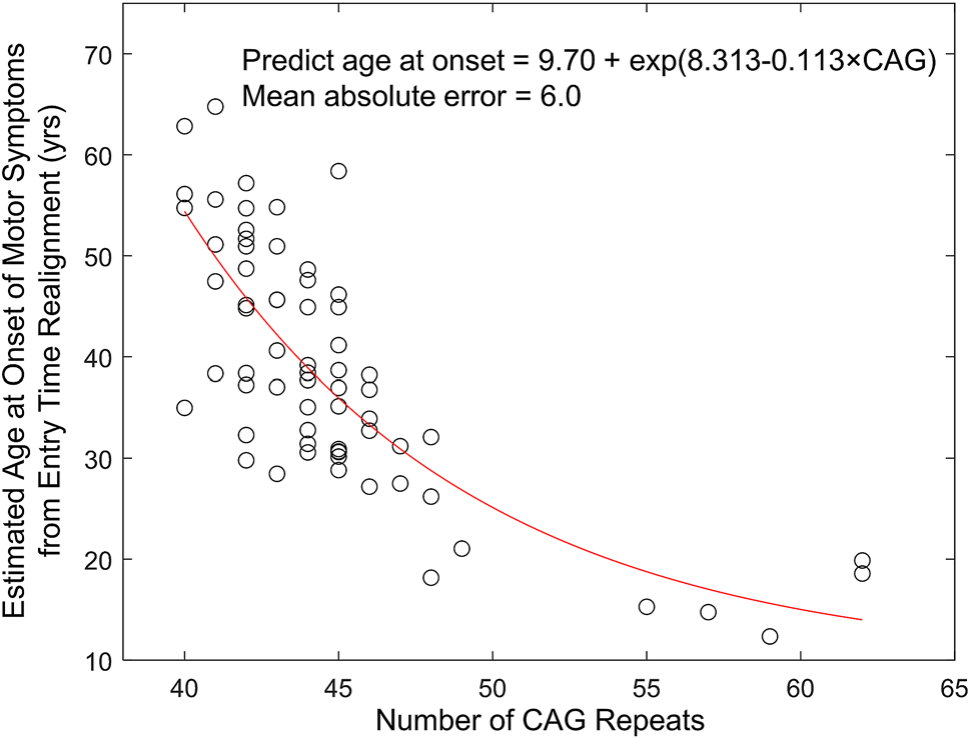
Estimated age at onset of motor symptoms based on the Bayesian entry time realignment method as a function of the number of CAG repeats in 66 patients. The red line represents the best fit of the model proposed by Langbehn et al. ($\mathrm{Age}\,\mathrm{at}\,\mathrm{onset}= a+ {\exp}\left\{b - c \times CAG\right\}$).

Since the increase of GI followed a second order model, we square root transformed GI and plotted the square root of GI as a function of time after enrollment in 71 patients (Fig. 6a). Different patients had various degrees of motor symptoms at the time of enrollment. By applying BETR with the assumption of different progression rates among different patients, we estimated the duration of symptoms (${\delta }_{i}$) for each patient and horizontally translated individual datasets by a factor of estimated ${\delta }_{i}$. This process converted the horizontal axis from time after enrollment to inferred duration of disease (Fig. 6b). On average, the square root of GI increased linearly at a mean (SE) rate of 0.46 (0.02) per year over 21.8 years (red line in Fig. 6b). The ICC between the estimated and observed square root of GI was 0.97 in this model (R^2^ = 0.93), with a mean difference of − 0.02 (Supplementary Fig. 5a,b). Based on this model, we estimated the mean ± SD age at onset of motor symptoms in Huntington’s disease as 38.5 ± 12.1 years, consistent with the literature^35^. At the patient-level, the estimated age at onset of motor symptoms decreased as a function of number of CAG repeats (66 patients; Fig. 7), consistent with a previously proposed formula by Langbehn et al. ($\mathrm{Age at onset}= a+ {\exp}\left\{b - c \times CAG\right\}$)^36^. In this function, CAG represents the number of CAG repeats which we obtained from primary authors of the study^23^; and *a*, *b*, and *c* are function parameters. We estimated the best fitted function as $\text{Predict age at onset} = 9.70 + \exp\left\{8.313-0.113\times CAG\right\}$, with a mean absolute error of 6.0 (Fig. 7).

When we assumed that all patients with Huntington’s disease had the same disease progression rates, individual datasets were fit onto a single trend line after we horizontally translated the datasets by a factor of estimated ${\delta }_{i}$ from BETR (Fig. 6c). This figure demonstrated the overall linear trend of square root of GI as a function of disease duration over two decades. Nevertheless, the “same progression rates” model had worse fit of patient-level data than the “different progression rates” model (DIC = 441 vs. 257; ICC = 0.95 vs. 0.97 in Supplementary Fig. 5a,c). Compared to the “different progression rates” model, the “same progression rates” model overestimated the square root of GI more when GI was small and underestimated the square root of GI more when GI was large (Supplementary Fig. 5b,d).

## Discussion

Using a hierarchical Bayesian regression modeling framework, we developed a novel statistical method, BETR, to reconstruct the long-term natural history of diseases based on data collected over short durations (Fig. 1). Compared to previous methods^5–22^, BETR allows for direct comparisons between hypothesized disease progression models through a well-established Bayesian model comparison tool (i.e., DIC)^24^. BETR also accounts for different disease progression rates across different patients, which enables estimations of patient-specific disease progression parameters, while correctly characterizing uncertainty during model estimation. Through extensive simulation studies, we demonstrated that BETR could identify the correct disease progression model among competing models nearly 100% of the time and was able to estimate patient-specific disease progression parameters with high accuracy and minimal bias (Table 1). By applying BETR in data from 318 eyes with GA (follow-up duration = 5.1 ± 3.0 years), a disease with a known long-term natural history model, we demonstrated that BETR identified the correct long-term progression model of GA area (i.e., the second order model) in real-world data. BETR showed that square root transformed GA area enlarged linearly over approximately 30 years but at different rates across different eyes (Fig. 4b). Application of BETR in Huntington’s disease, which had an unclear long-term natural history model, demonstrated that on average, the motor symptoms assessed by GI score increased quadratically over approximately 20 years until patients were chronically disabled (Fig. 6b).

BETR’s ability to allow variation in disease progression rates among individuals dramatically improves the model performance in both simulated and real-world data. It also improves the accuracy in estimating patient-level disease progression parameters. For example, GA is generally defined as a disease occurring in patients greater than 50 years^33,34^. In the “different progression rates” model, the estimated age at onset of GA in the example eye (56 years; solid blue circles in Fig. 4b) was reasonable. However, the estimated age at onset in the “same progression rates” model for the same eye was only 25 years (solid blue circles in Fig. 4c), far outside the normal range^33,34^. This was likely because the “same progression rates” model overestimated this eye’s baseline duration of disease. Nonetheless, the “same progression rates” model is still valid in inferring the “average” long-term natural history of a disease and can be helpful in visualizing the overall trend of the data especially when the data is noisy (Figs. 4c and 6c). It also has a computational advantage given that it includes fewer parameters than the “different progression rates” model, and will likely result in improved inference when there is no variability in the true progression rates. However, the flexibility of BETR allows for easy applications of both models, since the “same progression rates” models are a subset of BETR.

In the general disease progression model shown in Fig. 1, BETR makes three key assumptions about a disease (Table 2): First, all patients with the disease follow the same long-term disease progression model ($\mathrm{f}(t)$). Second, all patients with the disease have the same known disease severity (${\beta }_{0}$) at the time of disease onset, where ${\beta }_{0}$, measured by an observational parameter, usually remains 0 until there is detectable disease development. Third, the disease progression rate parameter (${\beta }_{1i}$) of a patient stays constant over time. Notably, $\mathrm{f}(t)$ does not need to be linear and can take different functional forms (e.g., first order, second order, and exponential model). For example, when $\mathrm{f}(t)$ is a second order model, ${\beta }_{1i}$ remains constant but the change of disease severity per year would change over time. The three assumptions are reasonable for many chronic diseases, including diseases in the present study (GA and Huntington’s disease) and diseases analyzed by previous entry time realignment methods (neovascular AMD^6–9^, Stargardt disease^15^, choroideremia^16,17^, drusen secondary to AMD^20^, Alzheimer’s disease^18^, fibrosing interstitial lung disease^19^). However, some diseases may not meet one or more assumptions so future authors should check the three assumptions before applying BETR to other diseases.

**Table 2:** Key assumptions of bayesian entry time realignment.

1. All patients with the same disease follow the same long-term disease progression model ($\mathrm{f}(t)$)	
2. All patients with the same disease have the same known disease severity (${\beta }_{0}$) at the time of disease onset	
3. The disease progression rate parameter (${\beta }_{1i}$) for a patient remains constant over time	

The model-based solution to entry time realignment that we propose with BETR allows for natural extensions to be added easily. Depending on the specific research question, future authors can modify the parameters in the general disease progression model in Fig. 1. For example, if future authors are interested in applying BETR to a disease in which ${\beta }_{0}$ is unknown and varies across patients (violating the second assumption of the current BETR method), we can treat it as unknown and specify a prior distribution for the parameter to allow the data to inform about its value. Future authors may also modify BETR to cluster disease progression parameters into severity groups, evaluate the impact of treatment or prognostic factors (e.g., demographics, genetics) on disease progression rates, or test more complicated disease progression models. Importantly, including additional unknown parameters will make the model more complicated and may require a large sample size to estimate the parameters accurately. Therefore, future studies should validate any further modifications of BETR through simulated and real-world data before applications.

Future authors may also apply BETR in fields outside medicine, where capturing the entire process is not attainable, time consuming, or cost prohibitive. For example, it is time consuming to image the entire cell cycle progression in certain cell types^37^. BETR may be used to infer the entire cell cycle progression process by synthesizing datasets collected from cells imaged over short periods. Similarly, BETR may also be applied to study the long-term degradation process of materials or other slow chemical reactions, which often take years or decades.

Results from the simulation studies provide an unbiased evaluation of BETR’s performance in various settings. Instead of arbitrarily defining simulation parameters, we used a real-world dataset (i.e., observed GA area from the AREDS) to generate simulated disease progression parameters that are free from user bias. Both simulated and observed data have similar mean follow-up duration (5 vs. 5.1 years) and mean number of visits (6 vs. 5.1). Comparison between the simulated data (Fig. 2b) and observed data (Fig. 6a) shows that the simulated data is comparable but noisier than the observed data. Also, the simulated data has smaller sample size than the observed data (100 vs. 318 eyes). BETR was still able to identify the correct disease progression model among competing models in 100 out of 100 simulations except for one scenario (Table 1). In 8 out of 100 simulations of the exponential model (same progression rates), BETR misidentified the second order model (different progression rates) as the best model likely due to noise in the data and the similarity between the second order and exponential functions over a short timeframe. The large noise of the data may also explain why the ICCs between estimated and true disease progression parameters were not perfect although they are still high (Table 1). Additionally, the small percentage differences (< 2%) between estimated and true mean duration of disease (${\delta }_{i}$) and disease progression rate (${\beta }_{1i}$) over 100 simulations demonstrate that BETR has minimal bias in estimating disease progression parameters (Table 1), indicating that BETR can estimate the mean ${\delta }_{i}$ and ${\beta }_{1i}$ very accurately when the same size is sufficient.

The BETR analysis of the long-term natural history of GA provides an empirical illustration of the strength and value of the method. GA is the advanced stage of non-exudative AMD, affecting over 5 million people worldwide^38^. Eyes with GA have atrophic lesions in the macula, and the area of GA lesions has been previously shown to enlarge quadratically over time^10,26–30^, which is also demonstrated clearly using BETR in the present study (ICC = 0.99; Fig. 4b). Due to the discovery of the long-term GA progression model, many clinical trials use the square root of GA area as an endpoint to evaluate treatment efficacy^26,39–42^, which can increase the statistical power^27,43^ and linearize the data for standard statistical analysis (e.g., linear mixed model)^32^. However, it is previously unclear if GA in a subset of patients follow a different disease progression model, which can represent a different GA phenotype with a distinct underlying pathophysiological mechanism. This question is difficult to answer because it is impractical to follow every eye with GA over decades and different models (e.g., the first order, second order, and exponential models) can appear very similar within a short timeframe. By performing BETR in randomly selected subsets, we can investigate this question and examine whether a subset prefers a different disease progression model. Interestingly, among 100 random subsampling rounds, the second order model performs the best in all 100 times (Fig. 3), which offers new evidence that GA area in most, if not all, eyes enlarge quadratically over time. Another importance of BETR in studying GA progression is the ability to estimate ages at onset of GA in individual eyes, which have been challenging to obtain because eyes with GA usually do not have visual symptoms in the first several years and present to clinics in later GA stages. BETR estimated the mean ± SD age at onset of GA in this cohort as 61.6 ± 7.3 years, consistent with findings from previous epidemiological studies^33,34^. Our patient-level data of the estimated age at onset of GA offer opportunities to investigate factors that affect the age at GA onset, which remain unclear^44^ and will be investigated in a future study.

BETR also provides insight into the long-term natural history of Huntington’s disease. Kuan et al. have proposed that GI score follows a linear progression in some patients but follows a quadratic progression in others^23^. These findings are consistent with our results from 100 random subsampling rounds, which show the first order model as the best model in 14 rounds and the second order model as the best model in 86 rounds. However, given the considerable noise of the clinical data and the similarity between the first and second order models over a short timeframe, we cannot conclude that different patients with Huntington’s disease follow different progression models without a larger sample size. Since a clinical trial aims to evaluate the efficacy of treatment on an entire patient cohort, the understanding of the best long-term disease progression model for the entire patient cohort is essential, and previously this has been unclear. Using BETR, we demonstrate the second order model as the best (i.e., lowest DIC) to describe the long-term disease natural history for the entire cohort, with a very high ICC of 0.97 (Fig. 6b). The estimated age at onset (38.5 ± 12.1 years) is also consistent with the literature (approximately 40 years)^35^, further supporting this model. On average, the square root of GI increases linearly over approximately 20 years from 0 (no symptoms) to 10 (all motor features). Future clinical trials may consider using the square root of GI as the endpoint to linearize the data to allow direct comparisons between patients at different disease stages.

Our study has several limitations. First, the current version of BETR requires existing hypothesis of disease progression models so that the algorithm can compare these models to identify the best version. However, the progression of some chronic diseases may follow a very complicated pattern so the long-term natural history of such diseases cannot be inferred reliably by BETR. For these diseases, future authors may attempt to use a spline function as the disease progression model in BETR, but further validations are required. Second, BETR can only be applied to a disease that meets the previously mentioned three key assumptions, although future extensions can be added to the existing framework for other applications. Third, we tested the first order, second order, and exponential models in our simulation studies because they are widely used in the literature to describe disease progression^10,15,16,18,20,23^ and can be challenging to differentiate within a short timeframe. However, other disease progression models exist, and we are unable to include all possible mathematical models in our simulations. Fourth, we generated simulated data based on real-world data (i.e., GA data from the AREDS). However, the simulated parameters may vary widely across different diseases and we were unable to simulate all possible settings, which include the number of patients, follow-up duration, follow-up interval between visits, disease progression models, mean and variance of baseline disease duration, mean and variance of disease progression rates, and mean and variance of measurement error. We encourage future authors to perform similar simulation studies with settings comparable to their data of interest.

In conclusion, we present BETR, a novel hierarchical Bayesian regression method, to investigate the long-term natural history of diseases using patient-level longitudinal data collected over short durations. We demonstrate through simulated and real-world data that the method can identify the correct long-term disease progression model among competing models and estimate patient-level disease progression parameters with high accuracy and low bias. By applying BETR in GA, we demonstrate that the square root of GA area enlarges linearly but at different rates among different eyes over approximately 30 years. Application of BETR in Huntington’s disease shows that, on average, the motor symptoms assessed by GI score increase quadratically over approximately 20 years until patients were chronically disabled. Future applications of BETR may advance the understanding of the long-term natural history of other chronic diseases as well as processes outside medicine.

## Methods

### Data

We obtained two datasets in the present study. The first dataset was the Age-Related Eye Disease Study (AREDS; ClinicalTrials.gov identifier, NCT00000145), containing color fundus photographs of patients with geographic atrophy (GA) secondary to age-related macular degeneration (AMD). We previously described the AREDS data acquisition and imaging analysis^14,43,45^. In brief, the AREDS was a prospective, multicenter, randomized controlled trial aiming to evaluate the efficacy of oral supplements on slowing the progression of age-related diseases, including AMD and cataract^46,47^. The study recruited 4757 participants aged 55 to 80 years from 1992 to 1998. We obtained raw AREDS data files (including medical data and color fundus photographs) via the database of Genotypes and Phenotypes (dbGaP Study Accession: phs000001.v3.p1) after receiving authorization for access^48^. The Yale University Institutional Review Board reviewed our study protocol and exempted the need of approval for our study. This study adhered to the tenets of the Declaration of Helsinki and all methods were performed in accordance with the Yale University Institutional Review Board's regulations. We extracted raw color fundus photographs of patients in the AREDS data files. We used ImageJ software (version 1.52p; National Institutes of Health, Bethesda, Maryland, USA)^49^ to manually delineate GA lesions in 1654 color fundus photographs (one image per visit) of 365 eyes in 247 patients in the AREDS database^45^. Based on our gradings, we measured the total area of GA at each visit of each eye. In the present analysis, we included 318 eyes that had gradable GA lesions in at least two visits^43^.

The second dataset we obtained was from a previously published retrospective study on Huntington’s disease by Kuan et al.^23^ Briefly, Kuan et al. collected data from patients who attended the Huntington’s disease clinic at the John van Geest Center for Brain Repair, UK, between 1995 and 2013. The motor impairments of patients with Huntington’s disease were assessed using the Unified Huntington’s Disease Rating Scale (UHDRS) total motor score. Kuan et al. transformed the UHDRS total motor score into generalized index (GI) score by deducting chorea and dystonia that have higher inter-rater variability and fluctuate over time. GI score ranges from 0 (no motor features) to 100 (all motor features)^23^. Also, Kuan et al. collected the demographic information and CAG repeat size (where available) of the patients. The Cambridge University Hospital NHS Foundation Trust approved their study. We obtained the de-identified data of 71 patients with Huntington’s disease directly from the primary authors of the study. We used GI score as the primary outcome measure of the present analysis.

### Bayesian entry time realignment method (BETR)

We developed BETR, a hierarchical Bayesian regression method, to investigate the long-term natural history of diseases using patient-level longitudinal data collected over short durations. The model assumes that the functional form of disease progression is the same for all patients, while allowing for variability in this relationship across patients through the introduction of person-specific regression parameters. Additionally, the method estimates a patient’s duration of disease at the time of enrollment, allowing us to leverage this information to better understand the long-term natural disease history without requiring long periods of follow up. The hypothesized model for disease severity is given as

1 $${Y}_{ij}-{\beta }_{0}={\beta }_{1i}\mathrm{f}\left({t}_{ij}+{\delta }_{i}\right){\epsilon }_{ij}; i=1,\dots ,n; j=1,\dots ,{m}_{i}$$

where ${Y}_{ij}$ represents disease severity for patient *i* (*n* total patients) during visit *j* (${m}_{i}$ visits for patient *i*) corresponding to continuous time ${t}_{ij}$ after enrollment. We work on the log-scale during model fitting given that disease severity is strictly positive such that

2 $$\mathrm{ln}\left({Y}_{ij}-{\beta }_{0}\right)=\mathrm{ln}\left({\beta }_{1i}\right)+\mathrm{ln}\left\{\mathrm{f}\left({t}_{ij}+{\delta }_{i}\right)\right\}+\mathrm{ln}({\epsilon }_{ij})\equiv {\beta }_{1i}^{*}+\mathrm{ln}\left\{\mathrm{f}\left({t}_{ij}+{\delta }_{i}\right)\right\}+{\epsilon }_{ij}^{*}$$

where ${\epsilon }_{ij}^{*}\sim \mathrm{N}\left(0,{\sigma }_{{\epsilon }^{*}}^{2}\right)$ represents the measurement error of disease severity and $\mathrm{N}(\mu , {\sigma }^{2})$ represents the univariate Gaussian distribution with mean $\mu$ and variance ${\sigma }^{2}$; ${\beta }_{0}$ represents the known disease severity at the time of disease onset, which is a constant for all patients; ${\beta }_{1i}^{*}$ represents the unknown patient-specific log disease progression rate (i.e., intercept parameter in Eq. (2)) which allows for patient-level variability in the disease severity relationship over time); $\mathrm{f}(t)$ describes the model for disease progression over time and is common across all patients (e.g., linear: $\mathrm{f}(t) = t$; quadratic: $\mathrm{f}(t)={t}^{2}$; exponential: $\mathrm{f}(t) = \mathrm{exp}\left\{t\right\}$); and ${\delta }_{i}>0$ is the unknown patient-specific duration of disease at the time of enrollment (i.e., alignment parameter).

We model the unknown log disease progression rate and log duration of disease parameters using hierarchical Bayesian methods such that

3 $${\beta }_{1i}^{*}\sim \mathrm{N}\left({\mu }_{{\beta }_{1}^{*}},{\sigma }_{{\beta }_{1}^{*}}^{2}\right)\,\mathrm{ and\,ln}\left({\delta }_{i}\right)\sim \mathrm{N}\left({\mu }_{\delta },{\sigma }_{\delta }^{2}\right)$$

respectively, with the remaining model parameters assigned weakly informative prior distributions of the form ${\sigma }_{{\epsilon }^{*}}^{2}, {\sigma }_{{\beta }_{1}^{*}}^{2}, {\sigma }_{\delta }^{2}\sim \mathrm{Inverse Gamma}(0.01, 0.01)$ and ${\mu }_{{\beta }_{1}^{*}},{\mu }_{\delta }\sim \mathrm{N}\left(0, {100}^{2}\right)$, allowing the data to drive the statistical inference rather than our prior beliefs. These intercept and alignment parameter distributions are centered around “global” mean values (${\mu }_{{\beta }_{1}^{*}}$ and ${\mu }_{\delta }$, respectively) with variances described by ${\sigma }_{{\beta }_{1}^{*}}^{2}$ and ${\sigma }_{\delta }^{2}$, all of which are estimated by the data. Expert prior information is most naturally incorporated into these “global” values (e.g., mean and variance) as they describe the average log disease progression rate and duration of disease at time of enrollment for a particular disease. However, patient-level variability may be more difficult to quantify, limiting the use of informative prior distributions for those corresponding parameters. In the case that there is no patient-level variability in these parameters, the variance terms can take on values near zero such that all intercept parameters are effectively equal to ${\mu }_{{\beta }_{1}^{*}}$ and all alignment parameters to $\mathrm{exp}\left\{{\mu }_{\delta }\right\}$.

For some diseases, the true progression model (i.e., $\mathrm{f}(t)$) will be known a priori. However, in settings where $\mathrm{f}(t)$ is unknown, we can fit BETR with different options for $\mathrm{f}(t)$ and use common Bayesian model comparison techniques to determine which specification best describes the long-term natural history of disease. Additionally, by estimating the person-specific disease progression rates and duration of disease, we can reconstruct the long-term natural course of disease for each patient and use the model for prediction of disease severity into the future.

Figure 1 shows the overview of applying the BETR method to investigate the long-term natural history of a disease using data from a clinical study of 4 example patients followed over short durations. At the time of study enrollment (left figure in Fig. 1), patients have different disease severities presumably because they enter the study at different time points of their individual disease courses (i.e., different ${\delta }_{i}$). To correct for the differences in participants’ entry time into the clinical study, we apply BETR to identify the best f(t) and to estimate ${\beta }_{i}^{*}$ and ${\delta }_{i}$ for each patient. We horizontally realign the entry times of the patients by a factor of ${\delta }_{i}$ onto the best $\mathrm{f}(t)$ function (right figure in Fig. 1), which essentially converts the horizontal axis from “elapsed time after enrollment” to “elapsed time after disease onset”, where “elapsed time after disease onset” = “elapsed time after enrollment” + ${\delta }_{i}$. In this way, we can reconstruct the long-term natural history of a disease by synthesizing data from patients followed over short durations.

### Entry time realignment model fitting

We implemented BETR in R 3.6.2 (R Foundation for Statistical Computing, Vienna, Austria) with the “rjags” package using Markov chain Monte Carlo sampling techniques^50^. For each fitted model, we collected 3000 total samples (1000 from 3 independent chains) from the joint posterior distribution of all model parameters after a burn-in period of 10,000 iterations per chain prior to convergence of the model. We further thinned the samples in each chain by a factor of 10, resulting in less correlated posterior samples. Posterior inference via posterior means and 95% quantile-based equal-tailed credible intervals was made using all 3000 samples. We formally tested the convergence of the models using Geweke’s diagnostic^31^. We applied BETR using different competing disease progression models (i.e., $\mathrm{f}(t)$) and calculated the deviance information criterion (DIC)^24^ for each. DIC is Bayesian model comparison tool that balances model fit and complexity, where a lower value indicates that the selected disease progression model is preferred^24^. We created code to simulate data from a disease progression model, fit our newly developed BETR method, and conduct posterior inference; available at https://github.com/warrenjl/Bayesian_ETR. We are currently working on creating a user-friendly R package for future implementations of BETR.

### Simulation study

We performed an extensive simulation study to investigate the ability of BETR in identifying the correct disease progression model ($\mathrm{f}(t)$) and in estimating patient-level disease progression parameters, including baseline duration of disease (${\delta }_{i}$) and disease progression rate (${\beta }_{1i}$). We generated simulated clinical trial data based on data from the 318 eyes with GA in the AREDS. In each simulated clinical trial, we predefined the ground truth $\mathrm{f}(t)$ as one of the following: (1) first order model (different progression rates), (2) second order model (different progression rates), (3) exponential model (different progression rates) (4) first order model (same progression rates), (5) second order model (same progression rates), and (6) exponential order model (same progression rates). The functional forms of the first order, second order, and exponential models were $f\left({t}_{ij}\right)={t}_{ij}$, $\mathrm{f}\left({t}_{ij}\right)={t}_{ij}^{2}$, and $\mathrm{f}\left({t}_{ij}\right)=\mathrm{exp}\left\{{t}_{ij}\right\}$, respectively. The disease progression rate, ${\beta }_{1i}$, was different among different patients in disease progression models (1) – (3) and was the same among different patients in models (4)–(6). Note that models (4) – (6) are limiting cases of models (1) – (3). Each simulated clinical trial dataset included 100 patients and each patient was followed annually over 5 years, similar to the GA data in the AREDS. In other words, ${t}_{ij}$ = 0, 1, 2, 3, 4, 5 years. We defined ${\beta }_{0}$ as 0 in the first and second order model. Since an exponential model would not reach 0, we defined ${\beta }_{0}$ as 0.05 (which was defined as the minimum GA size in several previous studies^51,52^) in the exponential model. We defined parameters (${\mu }_{{\beta }_{1}^{*}}, {\sigma }_{{\beta }_{1}^{*}}^{2}, {\mu }_{\delta },{\sigma }_{\delta }^{2}{, \mathrm{and} \sigma }_{{\epsilon }^{*}}^{2}$) in each $\mathrm{f}(t)$ for the purposes of simulating data by fitting the raw GA data in the AREDS using BETR with the corresponding $\mathrm{f}(t)$ and obtaining estimates from the model. Since all patients have the same progression rates in disease progression models (4)-(6), we defined ${\sigma }_{{\beta }_{1}^{*}}^{2}$ as 0 for these models. These values were then used for simulating ${\beta }_{1i}^{*}$ and ${\delta }_{i}$ from the models in (3). We summarized the parameters used in the simulated clinical studies in Supplementary Table 1. By using these parameters and the predefined $\mathrm{f}(t)$, we generated disease severity (${Y}_{ij}$) as a function of true time after disease onset (${t}_{ij}+{\delta }_{i}$) based on Eq. (2). Figure 2a shows an example of simulated ${Y}_{ij}$ as a function of (${t}_{ij}+{\delta }_{i}$) in 100 patients. Since ${\delta }_{i}$ is unknown in a typical clinical study, the data in a simulated clinical study was disease severity (${Y}_{ij}$) as a function of time after enrollment (${t}_{ij}$) (Fig. 2b as an example). In each simulated clinical study, we applied BETR on the simulated data with 4 competing $\mathrm{f}(t)$: models (1)–(3) and one of models (4)–(6) that has the same $\mathrm{f}(t)$ as the predefined ground truth model. For instance, if the ground truth $\mathrm{f}(t)$ is the second order model (different progression rates), we included models (1)-(3) and (5) as competing models. The $\mathrm{f}(t)$ that resulted in the lowest DIC was the best model selected by BETR. After identifying the best $\mathrm{f}(t)$, we compared the estimated ${\beta }_{1i}$ and ${\delta }_{i}$ with the true ${\beta }_{1i}$ and ${\delta }_{i}$ values (predefined in the simulation). We generated 100 simulated datasets for each ground truth $\mathrm{f}(t)$ model.

### BETR implementation in geographic atrophy

We used data from 318 eyes with GA in the AREDS to investigate whether our BETR algorithm could identify the correct disease progression model of a disease with a known natural history model. GA area was previously found to enlarge in a second order function ($\mathrm{f}\left({t}_{ij}\right)={t}_{ij}^{2}$)^10,26–30^. In this analysis, we used total area of GA lesions as the disease severity measurement^53^. We applied BETR to this dataset to compare the DIC of 4 competing disease progression models, including the first order model (different progression rates), second order model (different progression rates), exponential model (different progression rates), and second order model (same progression rates). The model with the lowest DIC was defined as the best model. We also estimated the ${\beta }_{1i}$ and ${\delta }_{i}$ in the best model using BETR. To account for the differences in participants’ entry time into the AREDS, we horizontally translated raw dataset by a factor of estimated ${\delta }_{i}$ to realign the patients and reconstruct the long-term natural history of GA. We compared the estimated and true GA size using R^2^ and intraclass correlation coefficient (ICC).

To investigate the hypothesis that most patients with GA follow the same progression model, we randomly selected 159 eyes and performed the BETR algorithm in this dataset. We repeated the random subsampling process and BETR analysis over 100 times to determine the number of times that each disease progression model was selected as the best model.

### BETR implementation in Huntington’s disease

We used data from 71 patients with Huntington’s disease to investigate the utility of BETR in a disease with an unclear natural history model. We used GI score^23^ as the primary outcome measure and applied BETR on this dataset in the same process as mentioned above. We randomly selected 50 patients for each random subsampling round and repeated the process for 100 times. We also estimated the age at onset of motor symptoms for each patient by subtracting estimated ${\delta }_{i}$ (estimated from the best fitting BETR model) from the patient’s age at the first visit. We then fitted estimated age at onset of motor symptoms as a function of number of CAG repeats for patients with CAG repeats greater than 35 using a model proposed by Langbehn et al. ($\mathrm{Age\,at\,onset}= a+ {\exp}\left\{b - c \times CAG\right\}$)^36^.

## Supplementary Information

Supplementary Information.
