# Supplementary material for: A hierarchical Bayesian entry time realignment method to study the long-term natural history of diseases
Source: Sci Rep. 2022 Mar 22;12:4869. doi: 10.1038/s41598-022-08919-1 (PMC8941125; doi:10.1038/s41598-022-08919-1)
Supplement: Supplementary file 1 — Supplementary Information. [file 41598_2022_8919_MOESM1_ESM.pdf]

Supplementary Information for “A Hierarchical Bayesian Entry Time Realignment Method to Study the Long-Term Natural History of Diseases”

Authors: Liangbo L. Shen,[1, 2] Lucian V. Del Priore,[2*] and Joshua L. Warren[3*]

Affiliations: [1]Department of Ophthalmology, University of California San Francisco, San Francisco, CA, USA

[2]Department of Ophthalmology and Visual Science, Yale University School of Medicine, New Haven, CT, USA

[3]Department of Biostatistics, Yale School of Public Health, New Haven, CT, USA

[*]**Corresponding Authors:** Lucian V. Del Priore, MD, PhD, Robert R. Young Professor and Chair, Department of Ophthalmology and Visual Science, Yale University School of Medicine, 40 Temple Street, Suite 1B, New Haven, CT 06510 (lucian.delpriore@yale.edu); Joshua L. Warren, PhD, Associate Professor, Department of Biostatistics, Yale School of Public Health, 350 George Street, New Haven, CT, 06511 (joshua.warren@yale.edu).

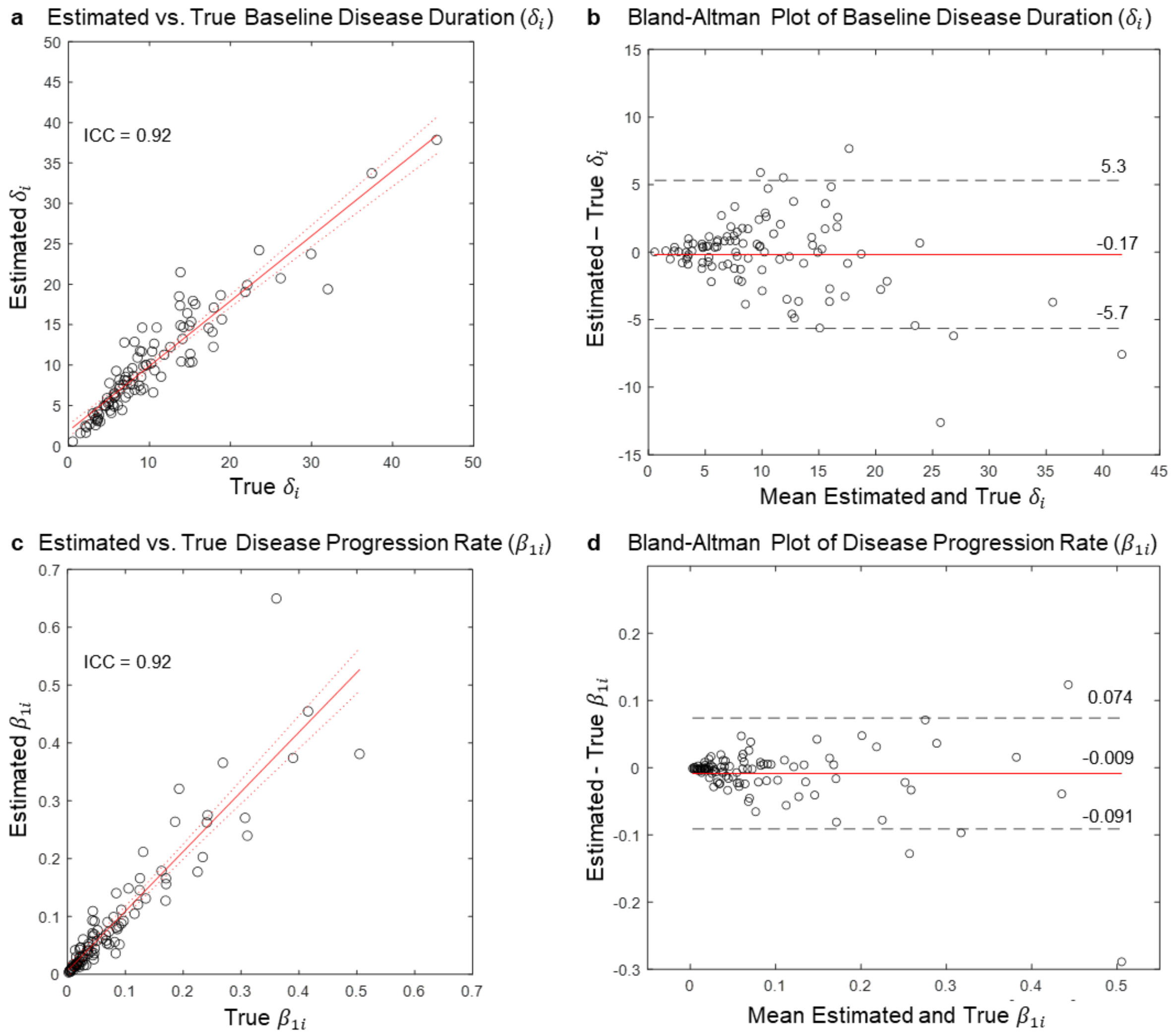

Supplementary Figure 1. Comparison between the estimated parameters from the Bayesian entry time realignment method and the true parameters generated in the simulation. ICC represents the intraclass correlation coefficient between the estimated and the true parameter. **a** Estimated versus true duration of disease at baseline (δ_i). The red dotted line represents the 95% confidence interval of the trend line (the solid red line). **b** Bland-Altman plot showing the difference between the estimated and true δ_i as a function of the mean between the 2 values. The red line represents the mean difference, and the dashed black lines represent the 95% limits of agreement. **c** Estimated versus true disease progression rate (β_{1i}). **d** Bland-Altman plot showing the difference between the estimated and true β_{1i} as a function of the mean between the 2 values.

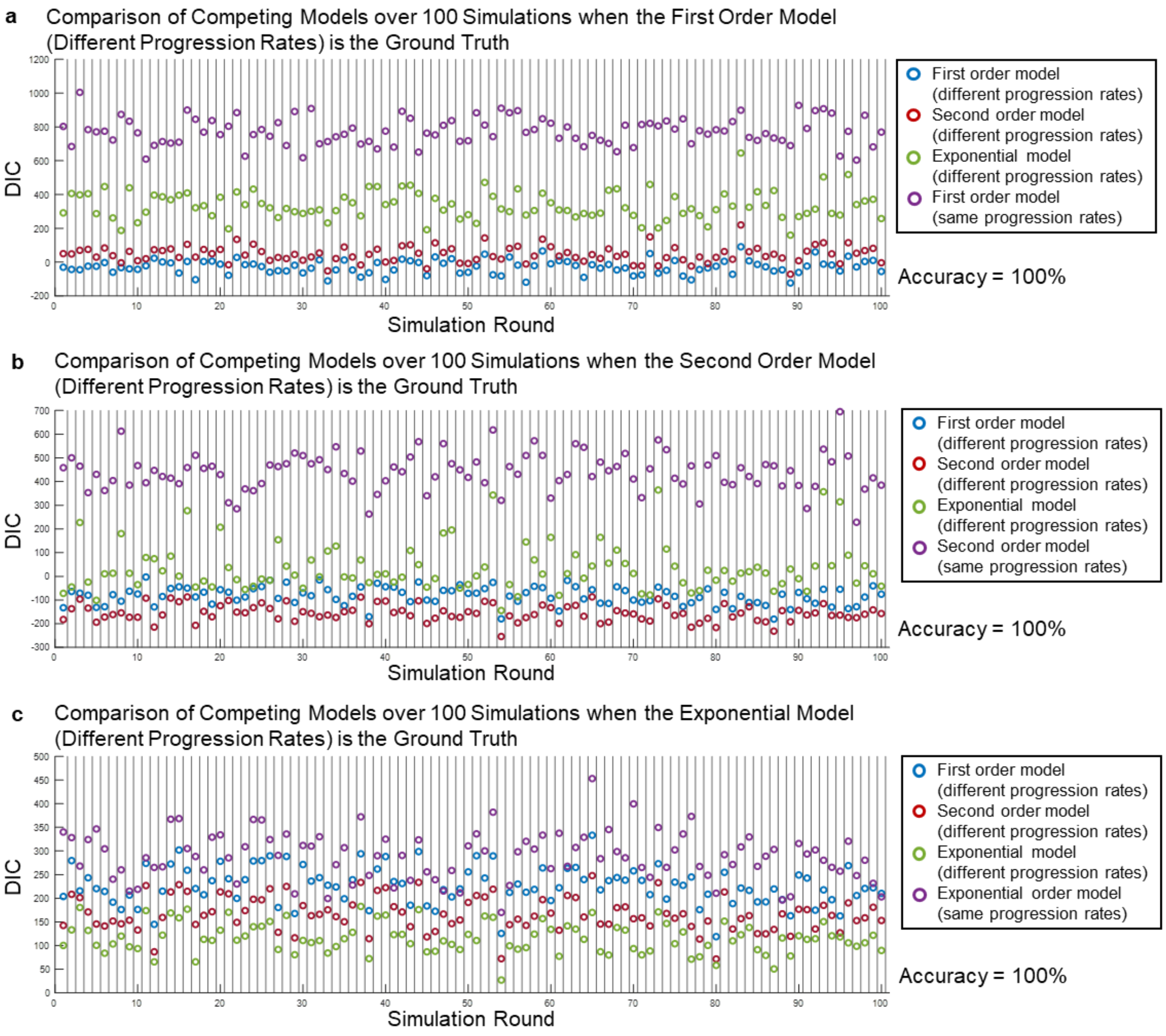

Supplementary Figure 2. Model performance when simulated patients have different disease progression rates. This figure shows deviance information criterion (DIC) of competing mathematical models over 100 simulations when the correct disease progression model is **a** first order, **b** second order, **c** exponential. The accuracy indicates the percentage of simulations when the entry time realignment algorithm determines the correct model as having the lowest DIC.

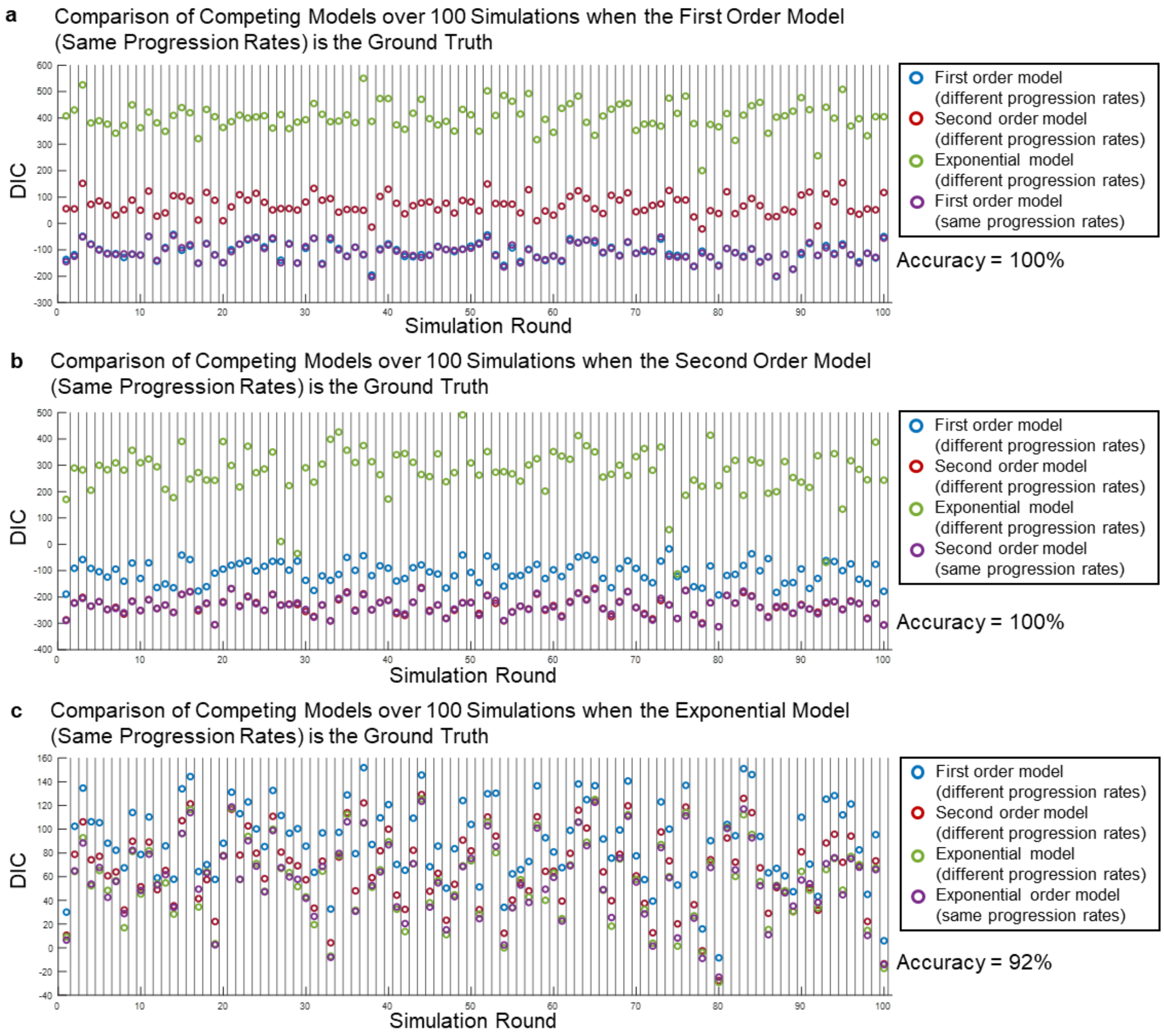

Supplementary Figure 3. Model performance when simulated patients have same disease progression rates. This figure shows deviance information criterion (DIC) of competing mathematical models over 100 simulations when the correct disease progression model is **a** first order, **b** second order, **c** exponential. The accuracy indicates the percentage of simulations when the entry time realignment algorithm determines the correct model (either “same progression rates” or “different progression rates”) as having the lowest DIC.

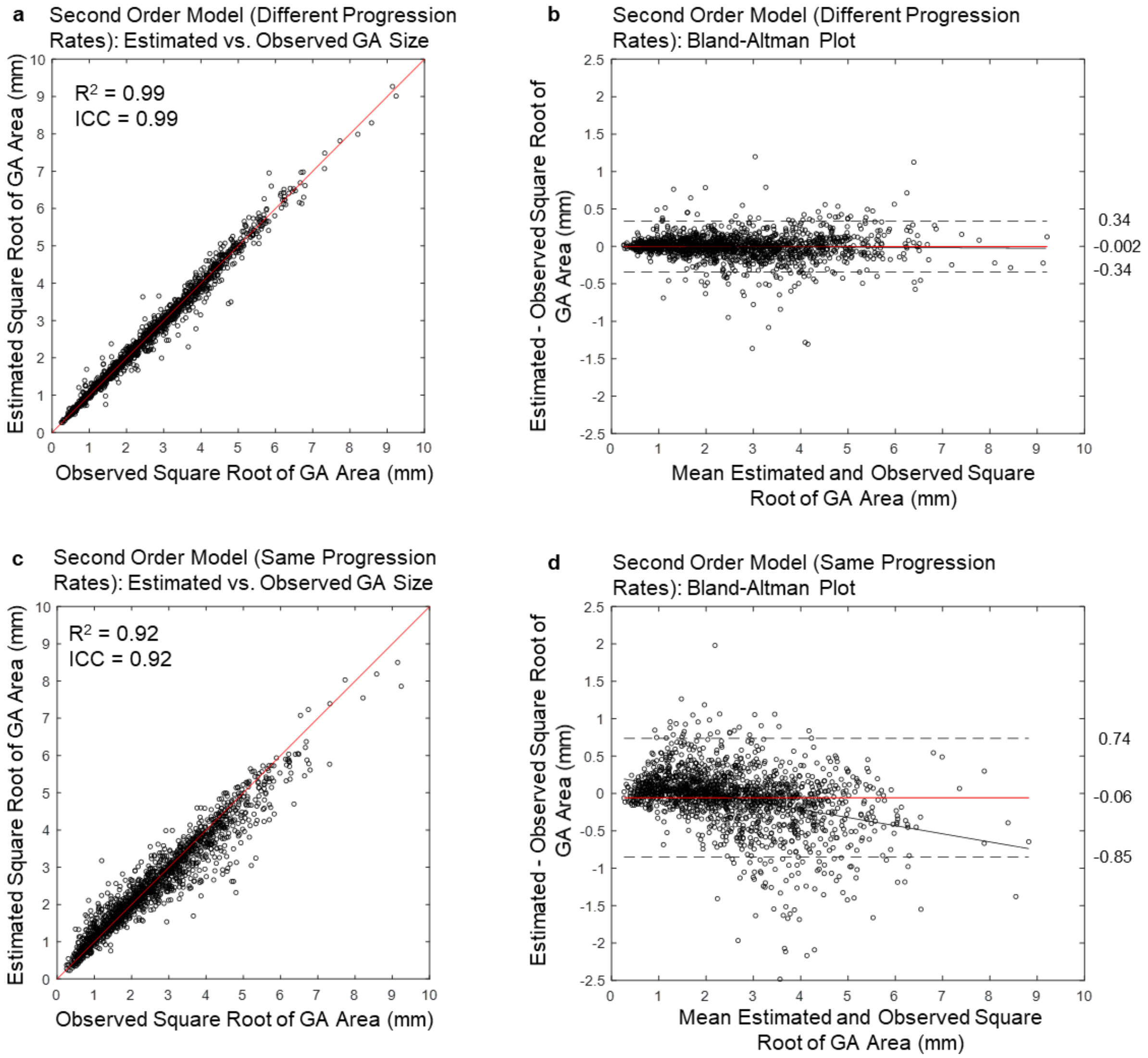

Supplementary Figure 4. Comparison between the observed square root of geographic atrophy (GA) area and the estimated square root of GA area from the Bayesian entry time realignment method (second order model) in eyes with GA. ICC represents the intraclass correlation coefficient between the estimated and the true square root of GA area. **a** Estimated versus the observed square root of GA area when we assumed different progression rates among different eyes. The red line represents the line of equivalence. **b** Bland-Altman plot showing the difference between the estimated and true square root of GA area when we assumed different progression rates among different eyes. The red line represents the mean difference, and the dashed black lines represent the 95% limit of agreement. The black solid line represents the linear fit of the data. **c** Estimated versus the observed square root of GA area when we assumed the same progression rates among all eyes. **d** Bland-Altman plot showing the difference between the estimated and true square root of GA area when we assumed the same progression rates among all eyes. The linear fit of the data (black line) suggests that this model overestimated the square root of GA area when GA size was small and underestimated the square root of GA area when GA size was large.

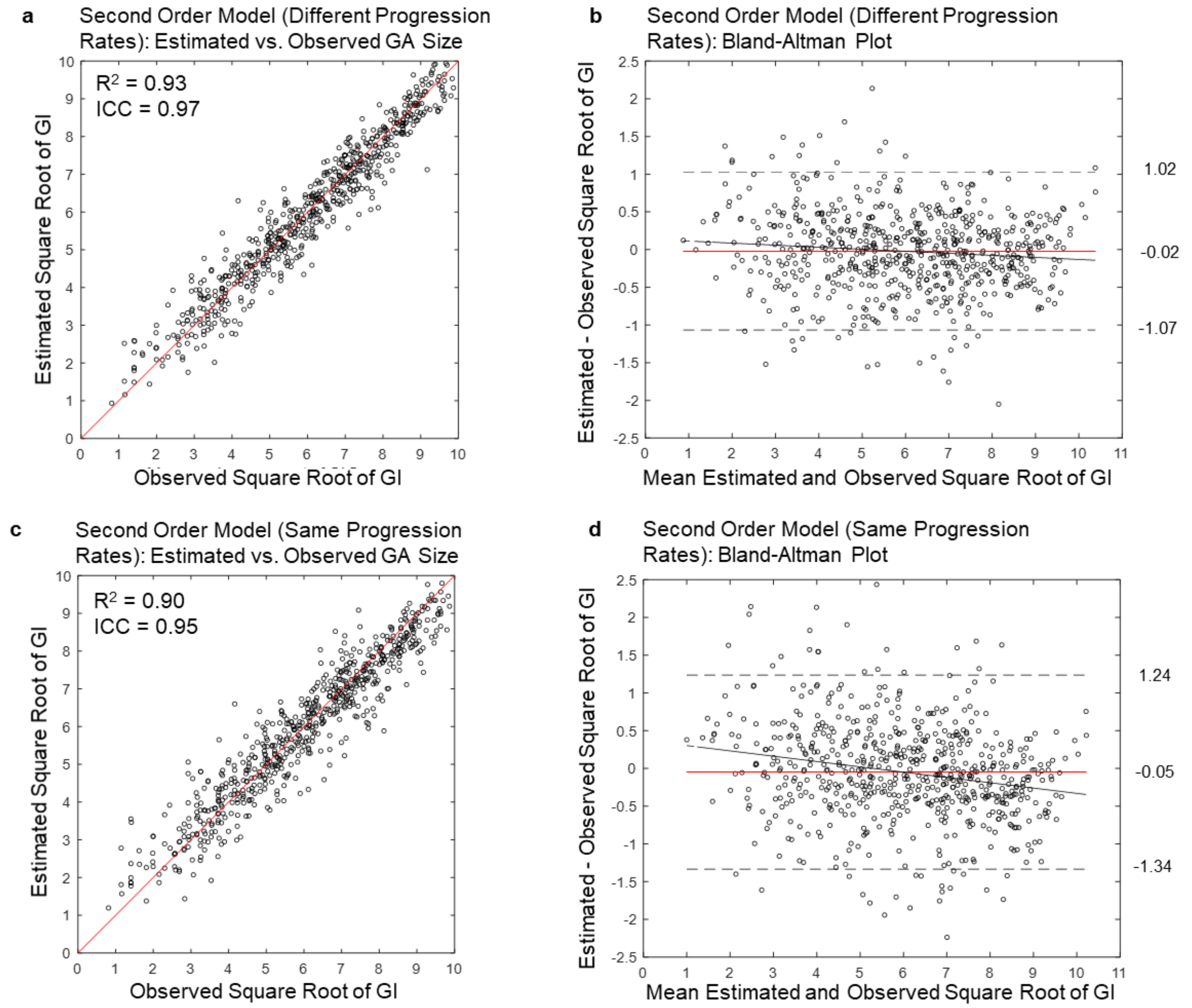

Supplementary Figure 5. Comparison between the observed square root of generalized index (GI) and the estimated square root of GI from the Bayesian entry time realignment method (second order model) in patients with Huntington's disease. ICC represents the intraclass correlation coefficient between the estimated and the true square root of GA area. **a** Estimated versus the observed square root of GI when we assumed different progression rates among different eyes. The red line represents the line of equivalence. **b** Bland-Altman plot showing the difference between the estimated and true square root of GI when we assumed different progression rates among different eyes. The red line represents the mean difference, and the dashed black lines represent the 95% limit of agreement. The black solid line represents the linear fit of the data. **c** Estimated versus the observed square root of GI when we assumed the same progression rates among all eyes. **d** Bland-Altman plot showing the difference between the estimated and true square root of GI when we assumed the same progression rates among all eyes. The linear fit of the data (black line) suggests that this model overestimated the square root of GI when GI was small and underestimated the square root of GI when GI was large.

Supplementary Table 1. Disease progression parameters used in simulation studies

Ground truth model	$\mu_{\beta_1^*}$	$\sigma^2_{\beta_1^*}$	μ_δ	σ^2_δ	$\sigma^2_{\epsilon^*}$
First order model (different progression rates)	-2.66	1.16	3.48	0.94	0.20
Second order model (different progression rates)	-8.07	1.26	4.46	0.68	0.18
Exponential model (different progression rates)	-4.09	0.39	5.39	0.57	0.23
First order model (same progression rates)	-2.66	0	3.48	0.94	0.20
Second order model (same progression rates)	-8.07	0	4.46	0.68	0.18
Exponential order model (same progression rates)	-4.09	0	5.39	0.57	0.23

$\mu_{\beta_1^*}$ = mean of the log disease progression rate; $\sigma^2_{\beta_1^*}$ = variance of the log disease progression rate; μ_δ = mean of log duration of disease; σ^2_δ = variance of log duration of disease; $\sigma^2_{\epsilon^*}$ = variance of the measurement error of disease severity.
